# Impact of *Eimeria tenella* Oocyst Dose on Parasite Replication, Lesion Score and Cytokine Transcription in the Caeca in Three Breeds of Commercial Layer Chickens

**DOI:** 10.3389/fvets.2021.640041

**Published:** 2021-02-22

**Authors:** Francesca Soutter, Dirk Werling, Sungwon Kim, Iván Pastor-Fernández, Virginia Marugán-Hernández, Fiona M. Tomley, Damer P. Blake

**Affiliations:** ^1^Department of Pathobiology and Population Sciences, Royal Veterinary College, London, United Kingdom; ^2^SALUVET, Animal Health Department, Faculty of Veterinary Sciences, Complutense University of Madrid, Madrid, Spain

**Keywords:** *Eimeria tenella*, chicken, dose, layer, lesion, qPCR, experimental optimisation

## Abstract

*Eimeria* species parasites infect the gastrointestinal tract of chickens, causing disease and impacting on production. The poultry industry relies on anticoccidial drugs and live vaccines to control *Eimeria* and there is a need for novel, scalable alternatives. Understanding the outcomes of experimental infection in commercial chickens is valuable for assessment of novel interventions. We examined the impact of different infectious doses of *Eimeria tenella* (one low dose, three high doses) in three commercial layer chicken lines, evaluating lesion score, parasite replication and cytokine response in the caeca. Groups of eight to ten chickens were housed together and infected with 250, 4,000, 8,000 or 12,000 sporulated oocysts at 21 days of age. Five days post-infection caeca were assessed for lesions and to quantify parasite replication by qPCR and cytokine transcription by RT-qPCR. Comparison of the three high doses revealed no significant variation between them in observed lesions or parasite replication with all being significantly higher than the low dose infection. Transcription of IFN-γ and IL-10 increased in all infected chickens relative to unchallenged controls, with no significant differences associated with dose magnitude (*p* > 0.05). No significant differences were detected in lesion score, parasite replication or caecal cytokine expression between the three lines of chickens. We therefore propose 4,000 *E. tenella* oocysts is a sufficient dose to reliably induce lesions in commercial layer chickens, and that estimates of parasite replication can be derived by qPCR from these same birds. However, more accurate quantification of *Eimeria* replication requires a separate low dose challenge group. Optimisation of challenge dose in an appropriate chicken line is essential to maximize the value of *in vivo* efficacy studies. For coccidiosis, this approach can reduce the numbers of chickens required for statistically significant studies and reduce experimental severity.

## Introduction

Coccidiosis, caused by protozoan parasites of the genus *Eimeria*, is a gastrointestinal disease commonly associated with chickens that has implications for chicken welfare and economic consequences for producers (1, 2). Seven *Eimeria* species are known to cause disease in chickens; each species infects a specific region of the gastrointestinal tract with variations in fecundity and pathogenicity noted between them (3).

Disease severity following *Eimeria* infection is parasite species- and dose-dependent (4, 5). Ingestion of a larger number of sporulated oocysts results in higher parasite loads in the gut and higher oocyst outputs, although fecundity reduces as dose size increases in a response known as the crowding effect (6). Other factors such as the host immune response, which is impacted by host nutrition, genetics and previous or concurrent infection, also contribute to variation in parasite replication and disease severity (7–9).

Whilst the influence of parasite dose on disease outcome has been well documented, much of the published work has used and compared inbred chicken lines or older, more traditional chicken breeds (8, 10, 11). There is little published on the impact of *Eimeria* dose on parasite replication or disease severity in modern commercial chicken lines, in particular layer hens. A wide numerical range of challenge doses are used to assess the efficacy of novel vaccines (12), with low doses (~100–250 oocysts) used to quantify parasite replication while minimizing the impact of the crowding effect, and high doses (~5,000–50,000 oocysts) used to evaluate protection against disease pathology (12). Both high and low dose challenges are important, although protection against disease pathology is often of primary interest to industry. However, calculating the parasite dose required to induce pathology within an ethical framework to minimize unnecessary suffering to experimental animals can be challenging.

In this study we have assessed a range of high dose parasite challenges in order to identify a dose that allows for quantification of both parasite replication and disease pathology. A low dose challenge was included for comparison, representing that commonly used for the assessment of parasite replication (13, 14). The commercial layer lines we selected are used worldwide and are thus more relevant than inbred lines for assessment of the efficacy of novel vaccines and anticoccidial treatments as they reflect a final target population for these products. Furthermore, variability in response to different *Eimeria* doses has not been documented between and within lines of many commercial layer chickens. Such information on variability is invaluable in determining appropriate experimental study sample sizes and for reproducible study design. Thus, for three commercial layer lines, we examined the impact of four *Eimeria tenella* oocyst doses by measuring four parameters that are commonly evaluated during anticoccidial vaccine studies *viz:* lesion score, parasite replication, transcription of interferon gamma (IFN-γ) (15) and transcription of interleukin (IL)-10 (15, 16).

## Materials and Methods

### Ethics Statement

This study was performed under a UK Home Office License according to the Animals (Scientific Procedures) Act 1986 (ASPA). Procedures were approved by the Royal Veterinary College (RVC) Animal Welfare Ethical Review Body (AWERB).

### Animals

Female Hy-line Brown, Hy-line Silver Brown and Lohmann Brown layer chickens were purchased from Hy-line UK Ltd. All chickens were vaccinated against Marek's disease (Nobilis Rismavac + CA126, MSD, Hoddesdon, UK) at the hatchery prior to the start of the study. Chickens were fed *ad libitum* using a commercial organic starter feed, free from anticoccidial drugs.

### Parasites

The *E. tenella* Houghton (H) strain was used throughout this study (17). Parasites were propagated by passage through chickens at the RVC, oocysts harvested as previously described (18), and were used within 1 month of sporulation.

### Experimental Design

Forty-five Hy-line Brown, 45 Hy-line Silver Brown and 45 Lohmann Brown female day of hatch chicks were weighed and divided into five groups of 27, containing 8–10 individuals of each line, which were split between two wire-floored cages per group. Chickens were weighed and then infected with sporulated *E. tenella* oocysts by oral gavage at 21 days of age. Chickens in group 1 received 250 oocysts, a low dose which can be used for quantification of parasite replication with minimal crowding. Group 2 received 4,000 oocysts, group 3 received 8,000 oocysts and group 4 received 12,000 oocysts; higher doses intended to induce pathology. Chickens in group 5 were not infected with *E. tenella*. Five days after *E. tenella* challenge (26 days of age) all birds were weighed and then killed humanely using an approved Schedule 1 method (cervical dislocation). The caeca were examined for lesion scoring as previously described (19). From each chicken, the left caeca was opened longitudinally, the luminal contents removed by scraping and immediately after lesion scoring the entire opened caeca was frozen in dry ice, prior to storage at −80°C. Right caeca were opened for lesion scoring and then discarded.

In a follow up vaccine study, a positive control group of 12 Hy-Line Brown female day of hatch chicks were weighed and housed in a wire-floored cage. Chickens were weighed and then infected with 4,000 sporulated *E. tenella* oocysts by oral gavage at 22 days of age. Six days after *E. tenella* challenge (28 days of age) all birds were weighed and then killed humanely using an approved Schedule 1 method (cervical dislocation). The caeca were examined for lesion scoring and treated and stored as described above.

### DNA and RNA Extraction

Genomic DNA (gDNA) and total RNA were extracted from thawed complete left caeca. Briefly, tissues were homogenized in Buffer RLT Plus (QIAGEN, Hilden, Germany) using a TissueRuptor homogenizer (QIAGEN) and nucleic acids extracted using the Allprep DNA/RNA extraction kit according to manufacturer's instructions. An optional DNase on-column digestion was performed to ensure no gDNA contamination of RNA. gDNA was treated with RNAse A (ThermoFisher, Waltham, MA, USA) and incubated at 37°C for 20 min to minimize RNA contamination of gDNA. gDNA and RNA quality and quantity were assessed by spectrophotometry using the DS-11 FX spectrophotometer (Denovix, Wilmington, DE, USA) and stored at −20 and −80°C, respectively.

### Quantitative PCR for Parasite Replication

Quantitative PCR for assessment of *E. tenella* genome copy number in caecal tissue was performed as previously described (20). Briefly gDNA purified from caecal tissue was amplified using previously published primers for *E. tenella* RAPD-SCAR marker Tn-E03-116. Primers for the chicken tata-binding protein (TBP) locus were used for normalization (Table 1). Absolute quantification was performed against a standard curve generated using serially diluted plasmid DNA, pGEM® T Easy plasmid (Promega, Madison, WI, USA) containing the amplicon of interest (EtenSCAR or ChickenTBP), to generate a standard curve ranging from 10^6^ copies to 10^1^ genome copies/ml. Parasite genome copy number was normalized by division with host (chicken) genome copy number per sample.

**Table 1:** Primers for measuring *Eimeria tenella* replication by qPCR and host cytokine expression by RT-qPCR.

**Gene**	**Primer**	**Direction**	**Primer sequence (5'-3')**	**Amplicon size (bp)**	**References**
**Parasite replication qPCR**					
*E.tenella* RAPD SCAR	Eten_qPCR	FOR	TCGTCTTTGGCTGGCTATTC	121	(21)
		REV	CAGAGAGTCGCCGTCACAGT		
Chicken TBP	TBP_qPCR	FOR	TAGCCCGATGATGCCGTAT	147	(22)
		REV	GTTCCCTGTGTCGCTTGC		
**RT-qPCR**					
Chicken TBP	TBP_RTqPCR	FOR	AGCTCTGGGATAGTGCCACAG	134	(23)
		REV	ATAATAACAGCAGCAAAACGCTTG		
Chicken SDHA	SDHA_RTqPCR	FOR	TCTGTCCATGGTGCTAATCG	126	(24)
		REV	TGGTTTAATGGAGGGGACTG		
Chicken β2M	B2M_RTqPCR	FOR	TACTCCGACATGTCCTTCAACG	150	(23)
		REV	TCAGAACTCGGGATCCCACTT		
Chicken RPL32	RPL32_RTqPCR	FOR	ATGGGAGCAACAAGAAGACG	139	(24)
		REV	TTGGAAGACACGTTGTGAGC		
Chicken GAPDH	GAPDH_RTqPCR	FOR	GAAGGCTGGGGCTCATCTG	150	(23)
		REV	CAGTTGGTGGTGCACGATG		
Chicken IL10		FOR	CATGCTGCTGGGCCTGAA		(15)
		REV	CGTCTCCTTGATCTGCTTGATG		
Chicken IFNG		FOR	GTGAAGAAGGTGAAAGATATCATGGA		(25)
		REV	GCTTTGCGCTGGATTCTCA		

Quantitative PCR was performed in triplicate in 20 μL reactions, containing 10 μL 2 × SsoFast™ EvaGreen® Supermix (Bio-Rad, Hercules, CA, USA), 1 μL of primers (3 μM FOR and 3 μM REV), 8 μL of molecular biology grade water (Invitrogen) and 1 μL of gDNA or water as a negative control. Hard-shelled 96-well reaction plates (Bio-Rad) were sealed with adhesive film (Bio-Rad) and loaded into a Bio-Rad CFX qPCR cycler. Reactions were heated to 95°C for 2 min, prior to 40 cycles consisting of 95°C for 15 s then 60°C for 30 s with a fluorescence reading taken after each cycle. Melting curve analysis was performed consisting of 15 s at 95°C, before cooling to 65°C for 60 s, then heating to 95°C in 0.5°C increments for 0.5 s.

### RT-qPCR for IFN-γ and IL-10 in the Caeca

Interferon gamma (IFN-γ) and interleukin 10 (IL-10) transcription in caecal tissue were measured as indicators of local cell mediated immune response. An Iscript™ cDNA synthesis kit (Bio-Rad) was used to prepare cDNA from 1 μg total RNA extracted from caecal tissue, according to manufacturer's instructions.

Primer sequences were based on previous publications (Table 1). Amplicons were assessed by melting peak analysis to confirm the presence of a single melting peak for each reaction. To determine PCR efficiency for each primer pair, standard curves were prepared using serial dilutions of purified PCR products. Curves were considered acceptable when the *R*^2^ value was >0.98 and primer efficiency considered acceptable when it was between 90 and 110%.

Quantitative PCR was used to quantify mRNA transcription (IFN-γ and IL-10 amplicons) in the caeca following *E. tenella* infection. Quantitative PCR was performed in triplicate in 20 μL reactions containing 10 μL 2 × SsoFast™ EvaGreen® Supermix (BioRad), 1 μL of primers (IFN-γ and IL-10:1.4 μM FOR and 1.4 μM REV; reference genes 2 μM FOR and 2 μM REV), 8 μL of molecular biology grade water (Invitrogen) and 1 μL of cDNA (test) or water (as a negative control). Hard-shelled white 96-well reaction plates (BioRad) were sealed with adhesive film (Bio-Rad) and loaded into a Bio-Rad CFX qPCR cycler. For all primer sets, reactions were heated to 95°C for 2 min, prior to 40 cycles consisting of 95°C for 15 s then 59°C (IFN-γ and IL-10) or 60°C (RPL32) for 30 s with a fluorescence reading taken after each cycle. Melting curve analysis was performed consisting of 15 s at 95°C, before cooling to 65°C for 60 s, then heating to 95°C in 0.5°C increments for 0.5 s.

The geNorm algorithm was used to identify the most suitable reference genes for normalization from a panel of five reference genes (TBP, SDHA, β2M, RPL32, and GAPDH; Table 1) tested on five samples that represented different individual chickens and different experimental groups.

The change in caecal gene transcription following infection with different *E. tenella* doses was determined by comparison of normalized expression (ΔΔCq) of genes of interest in caecal tissue. The mean Cq of each technical triplicate repeat, after amplification efficiency correction, was used. Where standard deviation (SD) between replicates was above 0.5, the assay was repeated for those samples. If following repeated analysis SD between replicates remained high, then samples were excluded from analysis. As a result, three samples (one from the 8,000 oocyst group, two from the 12,000 oocyst group) were excluded from the IFN-γ analysis and five samples (two from the uninfected controls, one from the 4,000 oocyst group, two from 8,000 oocyst group) were excluded from the IL-10 analysis. Determination of reference genes, normalization factors and normalized expression of targeted genes was performed using CFX Maestro™ Software (Bio-Rad).

### Statistical Analysis

Statistical analysis was carried out using GraphPad Prism 8 (Graph Software, LLC). One-way ANOVA was used to compare means of challenge groups and between chicken lines for weight gain, parasite replication and cytokine expression. Two-way ANOVA was performed to assess the impact of both challenge dose and chicken line. The Kruskal-Wallis test was used to compare ranked means of challenge groups and between groups for lesion scores. The *post-hoc* multiple comparison test used for all parameters was Tukeys. Spearmann rank correlation was used to assess correlations between parameters.

## Results

### Weights

All chickens were weighed on day of hatch, day of challenge (21 days of age) and at the end of the study (26 days of age). When individual chicken lines were evaluated separately, there were no significant differences in mean weight gain, pre- (D0–21) or post-challenge (D21–26) between any of the oocyst dose groups (Table 2). There were no significant differences in mean weight gain pre or post-challenge between dose groups after correction for multiple comparisons, even when all chicken lines were grouped together by dosage (*p* > 0.05). Lohmann brown chickens had significantly lower body weights than Hy-line Brown chickens at each time point (*p* < 0.05) and when evaluated by dose had lower body weight gain pre-challenge in the 12,000 challenge dose compared to the Hy-line Silver Browns and lower body weight gain post-challenge compared with Hy-line Browns in the post-challenge group (*p* < 0.05).

**Table 2:** Body weight gain in different chicken lines and the influence of *E. tenella* challenge.

**Chicken line**	**D0–D21: Pre- challenge mean weight gain (g) by dose (SD)**					**P value (ANOVA)**	**D21–D26: post- challenge mean weight gain (g) by dose (SD)**					***P* value (ANOVA)**
	**Unchallenged**	**250 oocyst**	**4000 oocyst**	**8000 oocyst**	**12000 oocyst**		**Unchallenged**	**250 oocyst**	**4000 oocyst**	**8000 oocyst**	**12000 oocyst**	
Lohmann Brown	168.4 (20.8)	174.7 (19.3)	167.1 (13.5)	176.3 (15.3)	160.3 (20.6)	0.44	72.2 (8.5)	77.5 (11.7)	74.7 (12.0)	71.0 (11.7)	65.4 (18.3)	0.4
Hy-line Brown	182.0 (15.4)	167.9 (17.2)	166.9 (22.1)	184.2 (19.4)	181.2 (12.6)	0.11	82.4 (7.9)	80.2 (4.8)	88.7 (10.6)	64.7 (13.5)	71.3 (6.8)	0.3
Hy-line Silver Brown	172.0 (13.9)	173.8 (24.0)	165.6 (15.6)	181.1 (5.1)	182.2 (17.5)	0.2	73.8 (6.8)	73.3 (5.7)	76.7 (13.3)	64.4 (21.4)	66.0 (22.0)	0.39
*P* value (ANOVA)	0.23	0.74	0.97	0.53	0.03^*^		0.02^**^	0.21	0.04^***^	0.65	0.73	

*SD, standard deviation*.

* *Lohmann Brown chickens significantly different from Hy-line Silver Brown after correction for multiple testing (Tukey's)*.

** *Lohmann Brown chickens significantly different from Hy-line Brown after correction for multiple testing (Tukey's)*.

*** *Not significant after correction for multiple testing*.

### Lesion Scores

**Figure 1:**
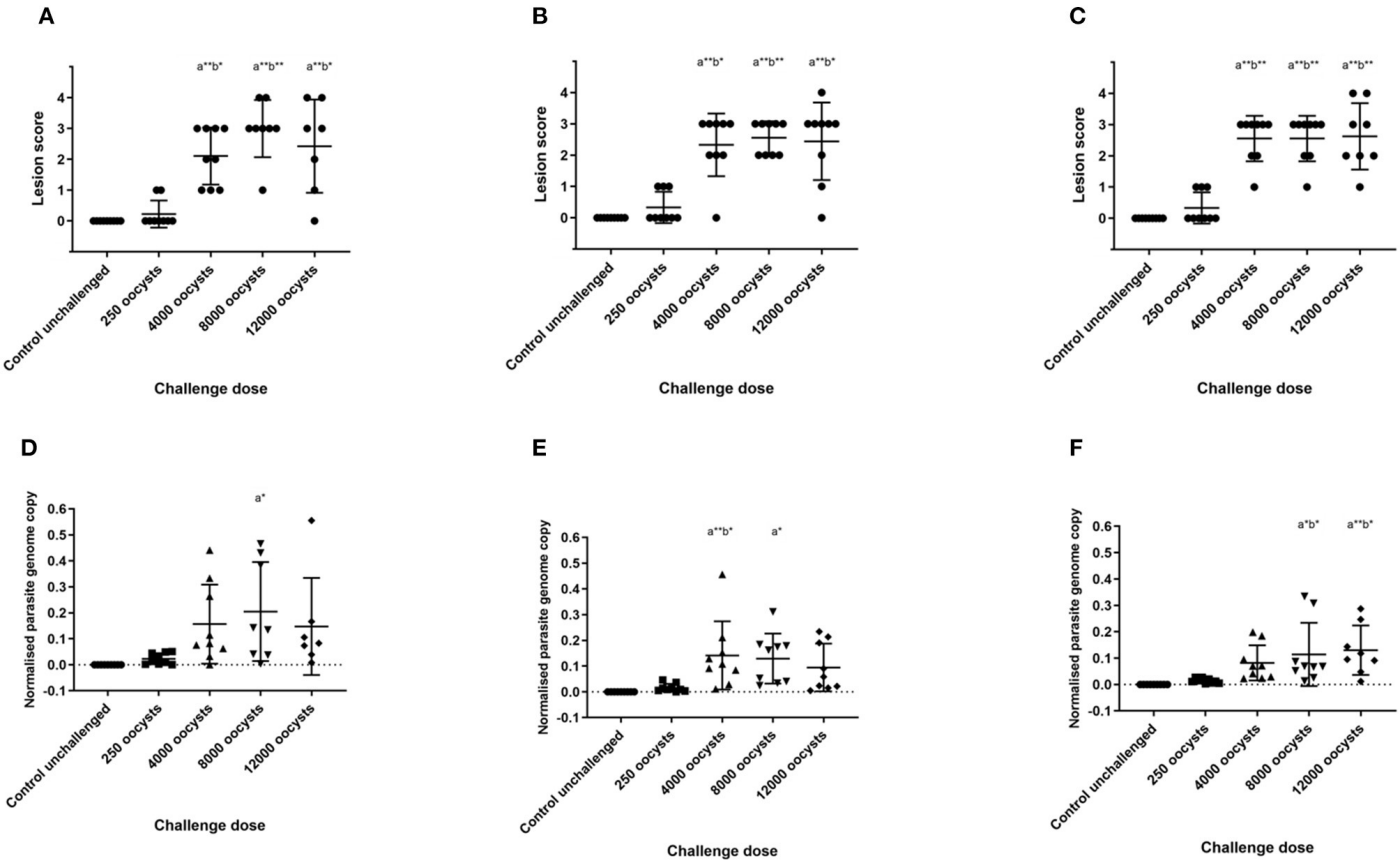
Lesion scores and parasite replication in caecal tissue measured by qPCR at 5 days post *E. tenella* infection in three layer chicken lines. Graphs **(A–C)** show lesion scores in chickens challenged with *E. tenella*. Each point demonstrates caecal lesion score of an individual bird 5 days post-challenge. Graphs **(D–F)** show parasite replication assessed by qPCR in chickens challenged with *E. tenella*. Each point demonstrates normalized parasite genome copy number (*E. tenella* gene copy/chicken TBP gene copy) from individual chickens 5 days post-challenge. Lines show the mean and standard deviation of each group. **(A,D)** show Lohmann Brown chickens, **(B,E)** show Hy-line Brown chickens, **(C,F)** show Hy-line Silver Brown chickens. Significant differences between means (One-way ANOVA and Tukeys correction) are shown; compared with control group (a) and with 250 oocyst dose (b), *p < 0.05, **p < 0.01.

Caecal lesions were evaluated and scored 5 days after *E. tenella* challenge (19). All chickens in the unchallenged groups scored zero. Average lesion score was higher in the 4,000 oocyst challenge dose than in the 250 oocyst dose for all chicken lines (Figures 1A–C). However, there was no further significant increase in mean ranked lesion score between the 4,000, 8,000, and 12,000 oocyst dose groups for any of the lines (*p* > 0.05), although there was more variability in lesion scores in the 12,000 oocyst dose group. Within each challenge dose group, there was no significant difference in lesion scores between the three layer lines evaluated (*p* > 0.05).

### Parasite Replication

Parasite replication in the caeca was assessed in all chickens using qPCR to measure *E. tenella* genome copies, and values were normalized against chicken genomic TBP gene copies. As with lesion score, mean normalized parasite genome number was higher in the 4,000 oocyst dose challenge group than in the 250 oocyst dose group (Figures 1D–F), but did not increase thereafter. There was no significant difference in mean parasite genomes between the different layer lines evaluated in each oocyst dose group (*p* > 0.05).

### RT-qPCR for IFN-γ and IL10

**Figure 2:**
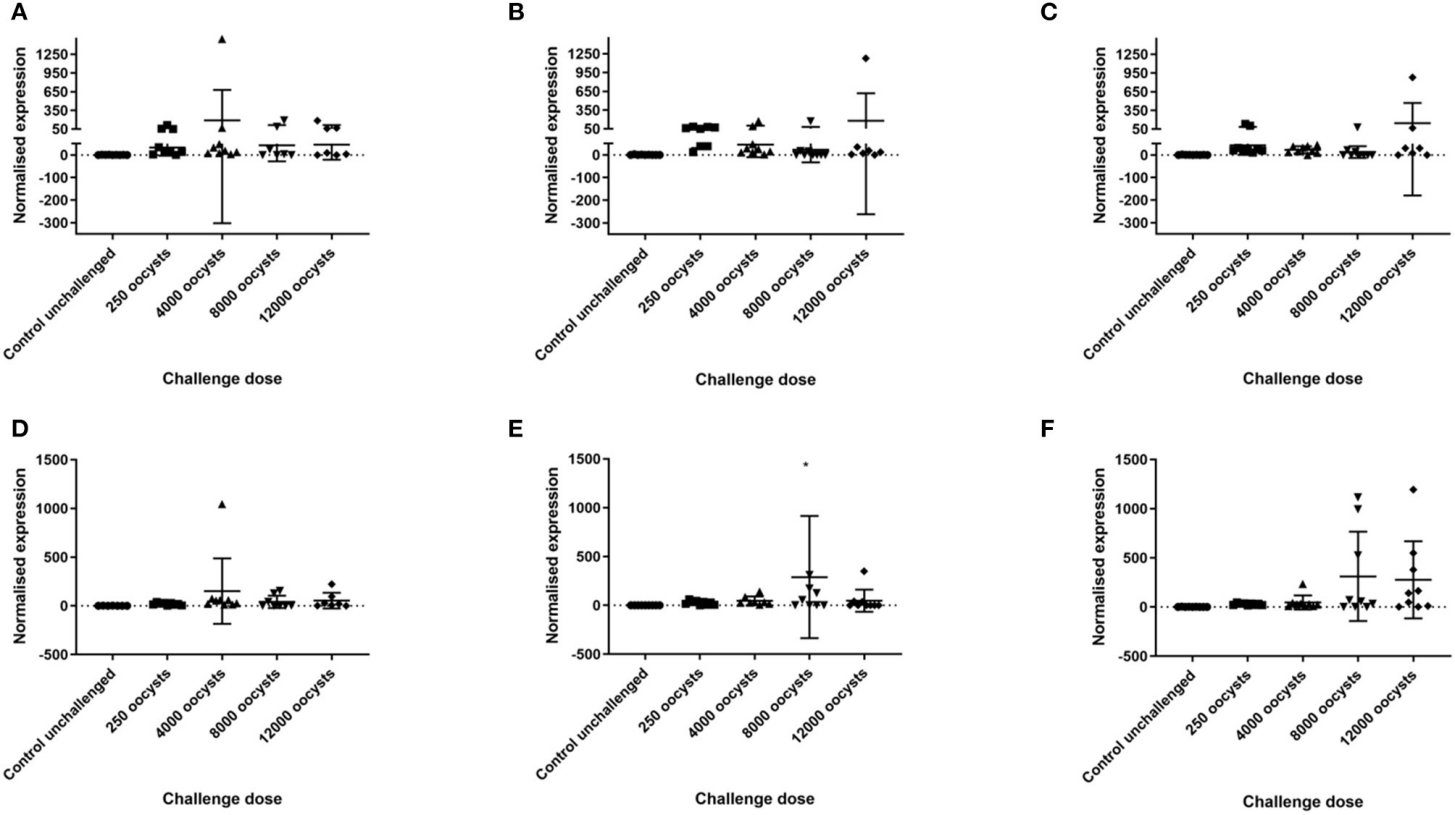
IFN-γ and IL-10 transcription in caecal tissue at 5 days post *E. tenella* infection in three layer chicken lines. Graphs **(A–C)** show relative normalized gene expression (ΔΔCq) of IFN-γ in caecal tissues from chickens challenged with *E. tenella*. Each point demonstrates transcription of IFN-γ, relative to zero, in the caeca 5 days post challenge. Graphs **(D–F)** show relative normalized gene expression (ΔΔCq) of IL-10 in caecal tissues from chickens challenged with *E. tenella*. Each point demonstrates transcription of IL-10, relative to zero, in the caeca 5 days post challenge. Lines show the mean and standard deviation of each group. **(A,D)** show Lohmann Brown chickens. **(B,E)** show Hy-line Brown chickens. **(C,F)** show Hy-line Silver Brown chickens. *One point in graph E is not displayed and had normalized gene expression above 1,500 (1,933.7).

Transcription of IFN-γ and IL-10 in the caeca at 5 days post infection was assessed by RT-qPCR. Based on the geNorm analysis, normalization was performed using the reference gene RPL32 (M value 0.33). There was no significant difference in normalized expression of IFN-γ between dose groups for individual chicken lines, or when all lines were grouped together. There was a significant increase in normalized IL-10 transcription in the caeca in chickens dosed with 8,000 oocysts compared with unchallenged controls and chickens challenged with 250 oocysts (*p* < 0.05) when all 3 lines were grouped together. However, when different layer lines were evaluated separately there was no significant difference in normalized transcription for IL-10 in the caeca in each oocyst dose group (*p* > 0.05) (Figure 2). There was no interaction between dose and breed on normalized expression of either IFN-γ or IL-10 (*p* > 0.05). It was noted that variation in normalized IL-10 transcription increased in the two Hy-line lines when challenged with 8,000 or 12,000 *E. tenella* oocysts.

### Correlations Between Parameters

Parasite replication in the caeca was positively correlated with lesion score (ρ 0.788, 95%CI 0.7098–0.8464, *p* < 0.01). IFN-γ and IL-10 transcription in the caeca were both positively correlated with parasite replication in the caeca (IFN-γ: ρ 0.239, 95%CI 0.06434–0.4003, *p* < 0.01; IL-10: ρ 0.368, 95%CI 0.2029–0.5130, *p* < 0.01). IL-10 transcription was also positively correlated with lesion score (ρ 0.194, 95%CI 0.01642–0.3604, *p* = 0.027). Post-challenge weight gain was inversely correlated with parasite replication (ρ −0.191, 95%CI −0.3553 to −0.01476, *p* = 0.029) and lesion score (ρ −0.262, 95%CI −0.4187 to −0.08913, *p* = 0.0025) and positively correlated with IFN-γ transcription (ρ 0.246, 95%CI 0.07146–0.4063, *p* = 0.005). Pre-challenge weight gain was not correlated with any other parameter (*p* > 0.05) (Supplementary Table 1).

## Discussion

Development of novel vaccines against coccidial parasites such as *E. tenella* requires well-designed vaccine studies that are robust and applicable to the commercial chicken population. This study sought to examine differences in outcome following different levels of high dose *E. tenella* oocyst challenge in three commercial layer chicken lines used worldwide, identifying an optimal dose level that reproducibly induces intestinal lesions. A low challenge dose widely used in experimental studies of *Eimeria* replication was included for comparison (13, 14). As anticipated, both parasite replication and lesion score were higher in chickens dosed with 4,000 *E. tenella* oocysts compared to those challenged with 250 oocysts. Increasing the oocyst dose above 4,000 did not result in higher parasite replication. The absence of notable variation in total parasite replication above a dose of 4,000 oocysts per chicken, despite a two- or three-fold increase in challenge, illustrates the “crowding effect” where increasing oocyst dose does not always increase oocyst output. Similarly, *E. maxima* replication was not significantly different in two broiler chicken lines when comparing doses of 2,500 and 7,000 oocysts (26). Other studies have suggested that peak replicative potential occurs at much lower oocyst doses than used here, 241 oocysts for *E. tenella* (6). Previously suggested explanations for the crowding effect include a lack of gut epithelial cells available to host parasite replication and the primary host immune response, possibly differing between more and less fecund *Eimeria* species (6, 27). Unexpectedly, increasing the oocyst dose above 4,000 did not result in an increase in caecal lesion score at 5 days post infection. Development of lesions in the caeca following *E. tenella* infection is complex and although at higher doses there was not a further increase in lesion severity, it is not possible to say whether this was the result of parasite crowding or a feature of the inflammatory immune response. There was an increase in variability in lesion score in the 12,000 oocyst dose group, as previously discussed development of lesions is complex and although this variability could be the result of variability in immune response between individual chickens the correlations between IFN—γ and IL-10 transcripts in the caeca and lesion score in our study were poor and cannot fully explain the variability. The lesion scores noted here are similar to the results of studies conducted in broiler chickens with *E. tenella* where lesion scores increased with increasing dose for all but the two highest doses, 10,000 and 100,000 oocysts (28). The dose titration described here suggests that an *E. tenella* dose of 4,000 oocysts per bird is sufficient to induce visible gross pathology in these commercial layer-type chickens, with no need to risk exposure to higher doses that may result in mortality.

**Figure 3:**
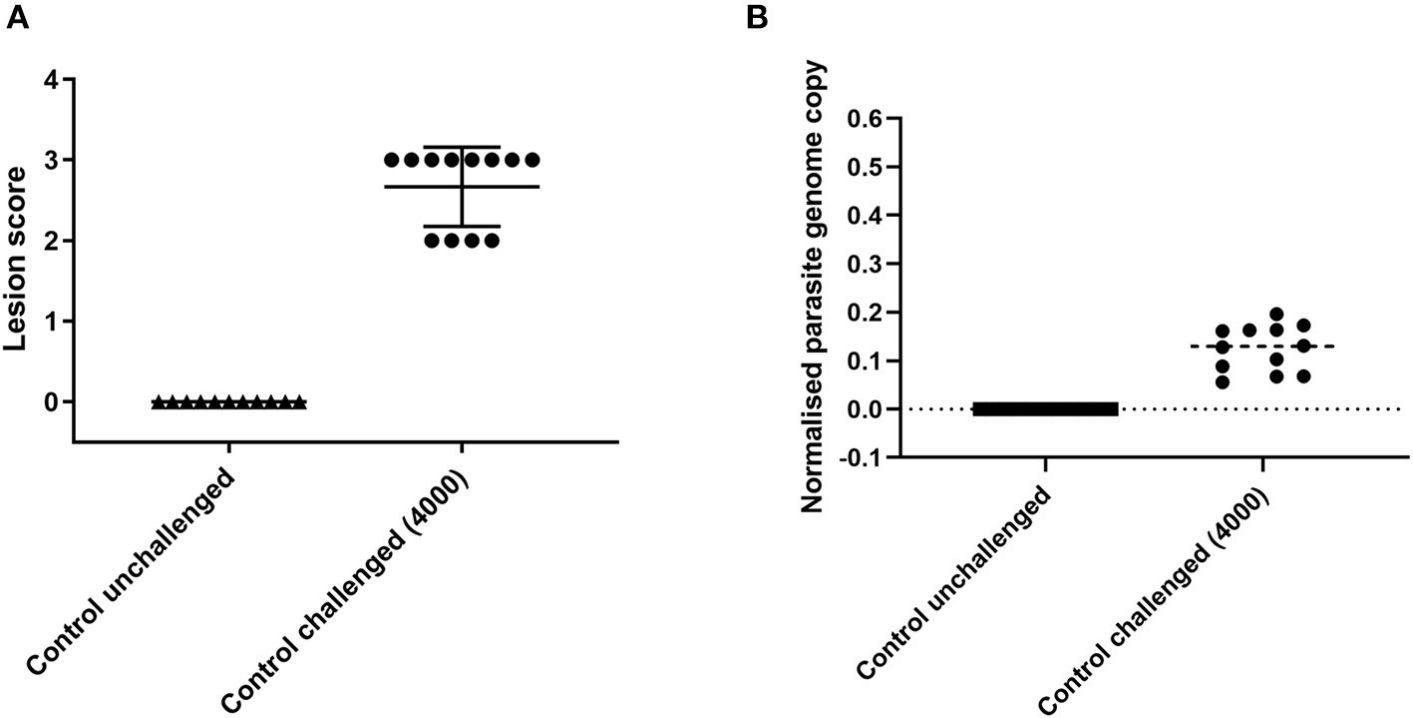
Lesion scores and parasite replication in caecal tissue measured by qPCR at 6 days post *E. tenella* infection in Hy-line brown chickens used as controls in a subsequent vaccine study. Graph **(A)** shows lesion scores in chickens challenged with *E. tenella*. Each point indicates the caecal lesion score from an individual chicken 6 days post-challenge. Graph **(B)** shows parasite replication assessed by qPCR in chickens challenged with *E. tenella*. Each point indicates the normalized parasite genome copy number (*E. tenella* gene copy/chicken TBP gene copy) from individual chickens 6 days post-challenge. Lines show the mean and standard deviation of each group.

Experimental reproducibility is important for comparison between successive trials, using different cohorts of chickens and different generations of parasites, is to be robust. Challenge of a second cohort of 12 female 3-week old Hy-line Brown chickens with 4,000 *E. tenella* H strain oocysts from a different parasite batch used as a positive control in a subsequent study and sampled at 6 days post infection revealed a comparable range of lesion scores (2–3; Figure 3A) and parasite replication (normalized parasite genome copy 0.06–0.2; Figure 3B).

Variation in *E. tenella* dose did not have a significant impact on body weight gain. Increasing levels of *E. tenella* infection have previously been associated with reduced body weight gain over a comparable timeframe in fast growing broiler chickens (29, 30), but this was not anticipated to be the case in the slower growing layer lines used in the present study. Chickens were sampled 5 days post-infection to permit accurate assessment of parasite replication and lesion score (19, 20). In order to assess impact on body weight gain, a second cohort of chickens could have been kept for a longer period of time, although observation of a significant difference would still not have been certain. Evaluation of other production measures that are relevant to layer chickens, such as time to point of lay, egg count and duration of laying, might be of interest, but there is little published support for association with coccidiosis in the peer reviewed literature and a much more prolonged study would have been required. Lohmann brown chickens had lower body weight at all time points compared to Hy-line chickens and had higher weight gain post challenge, this most likely reflects differences in genetic background and growth profiles between different chicken lines.

In this study increased variation was observed in IL-10, but not IFN-γ transcription in the caeca of chickens challenged with 8,000 or more oocysts (Figures 2D–F). Here, some but not all individuals transcribed IL-10 at higher levels when exposed to higher oocyst doses than chickens in the unchallenged or low dose challenge groups. Multiple studies have shown an increase in cytokine expression following *Eimeria* infection as a result of activation of lymphocytes due to infection and associated inflammation (15, 16, 31, 32). However, few studies have compared cytokine transcription or expression in chickens challenged with different doses and none have used commercial layer chickens. Indeed, only one study demonstrated results showing downregulation of IL-10 transcription in two broiler lines dosed with either 2,500 or 7,000 *E. maxima* oocysts, whilst IFN-γ was upregulated in both lines dosed with 2,500 oocysts and in one line dosed with 7,000 oocysts when compared with uninfected controls (33). This is different to the result of our study, where increased IL-10 transcription was noted in some individuals following a higher oocyst dose, possibly reflecting inherent genetic diversity within each hybrid line (29). Correlations of IFN-γ and IL-10 transcription with parasite replication, and of IL-10 with lesion score in our study were weak. Similarly weak associations have been found in other studies (7, 29), which suggests that they are not likely to be reliable markers of disease susceptibility.

Comparison between individual chickens exposed to the same oocyst dose revealed some variation in response to *E. tenella* infection, but no significant difference was detected between the three layer-lines assessed. Layer chickens are primarily produced by a small number of large companies, crossing three or more highly selected pedigree lines to produce the hybrid chickens used in many production systems. While these companies commonly produce different commercial layer “lines,” selection for performance traits is often very similar and one or more pedigree lines may be represented in the grandparental crosses used to produce multiple hybrid layer populations, possibly explaining the lack of variability observed (34). Evidence of resistance or susceptibility to *Eimeria* infection is apparent in some inbred chicken lines (35, 36) and native breeds such as the Egyptian Fayoumi chicken also appear more resistant compared with White Leghorns (37).

In conclusion, this study provides guidance regarding the optimal oocyst dose range for oral *E. tenella* challenge of commercial layer chickens to induce caecal lesions as a measure of parasite-induced pathology without excessively compromising chicken welfare. Specifically, we conclude that a dose of 4,000 sporulated oocysts of the *E. tenella* Houghton (H) strain per layer chicken is sufficient to accurately assess the impact of vaccination or chemoprophylaxis on pathology. Parasite replication can be assessed from the same individuals using quantitative PCR from caecal tissue, although it should be recognized that the *Eimeria* crowding effect may obscure small differences. More accurate assessment of parasite replication can be achieved using smaller parasite doses that are not appropriate for the assessment of pathology; however for this there is a requirement for duplicate cohorts – one low dose, one 4,000 dose, in challenge studies. The absence of significant differences in pathology and parasite replication between three commercial layer lines suggest that the results of studies in one line might be broadly applicable to the wider layer industry.

## Data Availability Statement

The raw data supporting the conclusions of this article will be made available by the authors, without undue reservation.

## Ethics Statement

The animal study was reviewed and approved by Royal Veterinary College (RVC) Animal Welfare Ethical Review Body (AWERB).

## Author Contributions

FS, DW, DB, and FT were involved in study conception and design and co-ordinated the experiments. FS, SK, IP-F, VM-H, and DB were involved in the *in vivo* study design and sample collection. FS performed the laboratory work, analyzed the data, and performed the statistical analysis. FS and DB wrote the manuscript with input from all the authors. All authors contributed to the article and approved the submitted version.

## Conflict of Interest

SK was employed by the company Touchlight Genetics Ltd., after completion of this work. The remaining authors declare that the research was conducted in the absence of any commercial or financial relationships that could be construed as a potential conflict of interest.
