# Supplementary material for: Impact of Eimeria tenella Oocyst Dose on Parasite Replication, Lesion Score and Cytokine Transcription in the Caeca in Three Breeds of Commercial Layer Chickens
Source: Front Vet Sci. 2021 Feb 22;8:640041. doi: 10.3389/fvets.2021.640041 (PMC7937735; doi:10.3389/fvets.2021.640041)
Supplement: Supplementary file 1 [file Table_1.docx]

***Supplementary Data***

***Table 1: Correlation matrix of parameters evaluated in E.tenella challenge study***

| Spearmann correlation(95% CI, p value) | | | | | | |
| --- | --- | --- | --- | --- | --- | --- |
|  | Parasite replication | Lesion score | IFN-γ | IL-10 | Weight-pre-challenge | Weight- post-challenge |
| Parasite replication | 1 | 0.79(0.71 - 0.85, <0.01) | 0.24(0.06 - 0.40, <0.01) | 0.37(0.20- 0.51, <0.01) | -0.04(-0.22- 0.13, 0.63) | -0.19(-0.35- -0.01, 0.03) |
| Lesion score | 0.79(0.71 - 0.85, <0.01) | 1 | 0.06(-0.11- 0.24, 0.46) | 0.19(0.016 - 0.36, 0.03) | 0.03(-0.14 - 0.21, 0.69) | -0.26(-0.42 - -0.09, <0.01) |
| IFN-γ | 0.24(0.06 - 0.40,0.01) | 0.06(-0.11 - 0.24,0.46) | 1 | 0.49(0.34 - 0.61, <0.01) | -0.17(-0.34 - 0.004, 0.05) | 0.25(0.071 - 0.41, <0.01) |
| IL-10 | 0.37(0.20- 0.51, <0.01) | 0.19(0.016 - 0.36, 0.03) | 0.49(0.34 - 0.61, <0.01) | 1 | 0.03(-0.15 - 0.21, 0.75) | 0.07(-0.11 - 0.24, 0.44) |
| Weight-pre-challenge | -0.04(-0.22- 0.13, 0.63) | 0.03(-0.14 - 0.21, 0.69) | -0.17(-0.34 - 0.004, 0.05) | 0.03(-0.15 - 0.21, 0.75) | 1 | 0.26(0.08 - 0.41, <0.01) |
| Weight- post-challenge | -0.19(-0.35- -0.01, 0.03) | -0.26(-0.42 - -0.09, <0.01) | 0.25(0.071 - 0.41, <0.01) | 0.07(-0.11 - 0.24, 0.44) | 0.26(0.08 - 0.41, <0.01) | 1 |
